# Impact of renal impairment on atrial fibrillation: ESC‐EHRA EORP‐AF Long‐Term General Registry

**DOI:** 10.1111/eci.13745

**Published:** 2022-01-17

**Authors:** Wern Yew Ding, Tatjana S. Potpara, Carina Blomström‐Lundqvist, Giuseppe Boriani, Francisco Marin, Laurent Fauchier, Gregory Y. H. Lip, the ESC‐EHRA EORP‐AF Long‐Term General Registry Investigators, G. Boriani, G.Y.H. Lip, L. Tavazzi, A. P. Maggioni, G‐A. Dan, T. Potpara, M. Nabauer, F. Marin, Z. Kalarus, L. Fauchier, A. Goda, G. Mairesse, T. Shalganov, L. Antoniades, M. Taborsky, S. Riahi, P. Muda, I. García Bolao, O. Piot, M. Nabauer, K. Etsadashvili, E. Simantirakis, M. Haim, A. Azhari, J. Najafian, M. Santini, E. Mirrakhimov, K.a Kulzida, A. Erglis, L. Poposka, M. Burg, H. Crijns, Ö. Erküner, D. Atar, R. Lenarczyk, M. Martins Oliveira, D. Shah, G‐A. Dan, E. Serdechnaya, T. Potpara, E. Diker, G.Y.H. Lip, D. Lane, E. Zëra, U. Ekmekçiu, V. Paparisto, M. Tase, H. Gjergo, J. Dragoti, A. Goda, M. Ciutea, N. Ahadi, Z. el Husseini, M. Raepers, J. Leroy, P. Haushan, A. Jourdan, C. Lepiece, L. Desteghe, J. Vijgen, P. Koopman, G. Van Genechten, H. Heidbuchel, T. Boussy, M. De Coninck, H. Van Eeckhoutte, N. Bouckaert, A. Friart, J. Boreux, C. Arend, P. Evrard, L. Stefan, E. Hoffer, J. Herzet, M. Massoz, C. Celentano, M. Sprynger, L. Pierard, P. Melon, B. Van Hauwaert, C. Kuppens, D. Faes, D. Van Lier, A. Van Dorpe, A. Gerardy, O. Deceuninck, O. Xhaet, F. Dormal, E. Ballant, D. Blommaert, D. Yakova, M. Hristov, T. Yncheva, N. Stancheva, S. Tisheva, M. Tokmakova, F. Nikolov, D. Gencheva, T. Shalganov, B. Kunev, M. Stoyanov, D. Marchov, V. Gelev, V. Traykov, A. Kisheva, H. Tsvyatkov, R. Shtereva, S. Bakalska‐Georgieva, S. Slavcheva, Y. Yotov, M. Kubíčková, A. Marni Joensen, A. Gammelmark, L. Hvilsted Rasmussen, P. Dinesen, S. Riahi, S. Krogh Venø, B. Sorensen, A. Korsgaard, K. Andersen, C. Fragtrup Hellum, A. Svenningsen, O. Nyvad, P. Wiggers, O. May, A. Aarup, B. Graversen, L. Jensen, M. Andersen, M. Svejgaard, S. Vester, S. Hansen, V. Lynggaard, M. Ciudad, R. Vettus, P. Muda, A. Maestre, S. Castaño, S. Cheggour, J. Poulard, V. Mouquet, S. Leparrée, J. Bouet, J. Taieb, A. Doucy, H. Duquenne, A. Furber, J. Dupuis, J. Rautureau, M. Font, P. Damiano, M. Lacrimini, J. Abalea, S. Boismal, T. Menez, J. Mansourati, G. Range, H. Gorka, C. Laure, C. Vassalière, N. Elbaz, N. Lellouche, K. Djouadi, F. Roubille, D. Dietz, J. Davy, M. Granier, P. Winum, C. Leperchois‐Jacquey, H. Kassim, E. Marijon, J. Le Heuzey, J. Fedida, C. Maupain, C. Himbert, E. Gandjbakhch, F. Hidden‐Lucet, G. Duthoit, N. Badenco, T. Chastre, X. Waintraub, M. Oudihat, J. Lacoste, C. Stephan, H. Bader, N. Delarche, L. Giry, D. Arnaud, C. Lopez, F. Boury, I. Brunello, M. Lefèvre, R. Mingam, M. Haissaguerre, M. Le Bidan, D. Pavin, V. Le Moal, C. Leclercq, O. Piot, T. Beitar, I. Martel, A. Schmid, N. Sadki, C. Romeyer‐Bouchard, A. Da Costa, I. Arnault, M. Boyer, C. Piat, L. Fauchier, N. Lozance, S. Nastevska, A. Doneva, B. Fortomaroska Milevska, B. Sheshoski, K. Petroska, N. Taneska, N. Bakrecheski, K. Lazarovska, S. Jovevska, V. Ristovski, A. Antovski, E. Lazarova, I. Kotlar, J. Taleski, L. Poposka, S. Kedev, N. Zlatanovik, S. Jordanova, T. Bajraktarova Proseva, S. Doncovska, D. Maisuradze, A. Esakia, E. Sagirashvili, K. Lartsuliani, N. Natelashvili, N. Gumberidze, R. Gvenetadze, K. Etsadashvili, N. Gotonelia, N. Kuridze, G. Papiashvili, I. Menabde, S. Glöggler, A. Napp, C. Lebherz, H. Romero, K. Schmitz, M. Berger, M. Zink, S. Köster, J. Sachse, E. Vonderhagen, G. Soiron, K. Mischke, R. Reith, M. Schneider, W. Rieker, D. Boscher, A. Taschareck, A. Beer, D. Oster, O. Ritter, J. Adamczewski, S. Walter, A. Frommhold, E. Luckner, J. Richter, M. Schellner, S. Landgraf, S. Bartholome, R. Naumann, J. Schoeler, D. Westermeier, F. William, K. Wilhelm, M. Maerkl, R. Oekinghaus, M. Denart, M. Kriete, U. Tebbe, T. Scheibner, M. Gruber, A. Gerlach, C. Beckendorf, L. Anneken, M. Arnold, S. Lengerer, Z. Bal, C. Uecker, H. Förtsch, S. Fechner, V. Mages, E. Martens, H. Methe, T. Schmidt, B. Schaeffer, B. Hoffmann, J. Moser, K. Heitmann, S. Willems, S. Willems, C. Klaus, I. Lange, M. Durak, E. Esen, F. Mibach, H. Mibach, A. Utech, M. Gabelmann, R. Stumm, V. Ländle, C. Gartner, C. Goerg, N. Kaul, S. Messer, D. Burkhardt, C. Sander, R. Orthen, S. Kaes, A. Baumer, F. Dodos, A. Barth, G. Schaeffer, J. Gaertner, J. Winkler, A. Fahrig, J. Aring, I. Wenzel, S. Steiner, A. Kliesch, E. Kratz, K. Winter, P. Schneider, A. Haag, I. Mutscher, R. Bosch, J. Taggeselle, S. Meixner, A. Schnabel, A. Shamalla, H. Hötz, A. Korinth, C. Rheinert, G. Mehltretter, B. Schön, N. Schön, A. Starflinger, E. Englmann, G. Baytok, T. Laschinger, G. Ritscher, A. Gerth, D. Dechering, L. Eckardt, M. Kuhlmann, N. Proskynitopoulos, J. Brunn, K. Foth, C. Axthelm, H. Hohensee, K. Eberhard, S. Turbanisch, N. Hassler, A. Koestler, G. Stenzel, D. Kschiwan, M. Schwefer, S. Neiner, S. Hettwer, M. Haeussler‐Schuchardt, R. Degenhardt, S. Sennhenn, S. Steiner, M. Brendel, A. Stoehr, W. Widjaja, S. Loehndorf, A. Logemann, J. Hoskamp, J. Grundt, M. Block, R. Ulrych, A. Reithmeier, V. Panagopoulos, C. Martignani, D. Bernucci, E. Fantecchi, I. Diemberger, M. Ziacchi, M. Biffi, P. Cimaglia, J. Frisoni, G. Boriani, I. Giannini, S. Boni, S. Fumagalli, S. Pupo, A. Di Chiara, P. Mirone, E. Fantecchi, G. Boriani, F. Pesce, C. Zoccali, V.L. Malavasi, A. Mussagaliyeva, B. Ahyt, Z. Salihova, K. Koshum‐Bayeva, A. Kerimkulova, A. Bairamukova, E. Mirrakhimov, B. Lurina, R. Zuzans, S. Jegere, I. Mintale, K. Kupics, K. Jubele, A. Erglis, O. Kalejs, K. Vanhear, M. Burg, M. Cachia, E. Abela, S. Warwicker, T. Tabone, R. Xuereb, D. Asanovic, D. Drakalovic, M. Vukmirovic, N. Pavlovic, L. Music, N. Bulatovic, A. Boskovic, H. Uiterwaal, N. Bijsterveld, J. De Groot, J. Neefs, N. van den Berg, F. Piersma, A. Wilde, V. Hagens, J. Van Es, J. van Opstal, B. Van Rennes, H. Verheij, W. Breukers, G. Tjeerdsma, R. Nijmeijer, D. Wegink, R. Binnema, S. Said, Ö. Erküner, S. Philippens, W. van Doorn, H. Crijns, T. Szili‐Torok, R. Bhagwandien, P. Janse, A. Muskens, M. van Eck, R. Gevers, N. van der Ven, A. Duygun, B. Rahel, J. Meeder, A. Vold, C. Holst Hansen, I. Engset, D. Atar, B. Dyduch‐Fejklowicz, E. Koba, M. Cichocka, A. Sokal, A. Kubicius, E. Pruchniewicz, A. Kowalik‐Sztylc, W. Czapla, I. Mróz, M. Kozlowski, T. Pawlowski, M. Tendera, A. Winiarska‐Filipek, A. Fidyk, A. Slowikowski, M. Haberka, M. Lachor‐Broda, M. Biedron, Z. Gasior, M. Kołodziej, M. Janion, I. Gorczyca‐Michta, B. Wozakowska‐Kaplon, M. Stasiak, P. Jakubowski, T. Ciurus, J. Drozdz, M. Simiera, P. Zajac, T. Wcislo, P. Zycinski, J. Kasprzak, A. Olejnik, E. Harc‐Dyl, J. Miarka, M. Pasieka, M. Ziemińska‐Łuć, W. Bujak, A. Śliwiński, A. Grech, J. Morka, K. Petrykowska, M. Prasał, G. Hordyński, P. Feusette, P. Lipski, A. Wester, W. Streb, J. Romanek, P. Woźniak, M. Chlebuś, P. Szafarz, W. Stanik, M. Zakrzewski, J. Kaźmierczak, A. Przybylska, E. Skorek, H. Błaszczyk, M. Stępień, S. Szabowski, W. Krysiak, M. Szymańska, J. Karasiński, J. Blicharz, M. Skura, K. Hałas, L. Michalczyk, Z. Orski, K. Krzyżanowski, A. Skrobowski, L. Zieliński, M. Tomaszewska‐Kiecana, M. Dłużniewski, M. Kiliszek, M. Peller, M. Budnik, P. Balsam, G. Opolski, A. Tymińska, K. Ozierański, A. Wancerz, A. Borowiec, E. Majos, R. Dabrowski, H. Szwed, A. Musialik‐Lydka, A. Leopold‐Jadczyk, E. Jedrzejczyk‐Patej, M. Koziel, R. Lenarczyk, M. Mazurek, Z. Kalarus, K. Krzemien‐Wolska, P. Starosta, E. Nowalany‐Kozielska, A. Orzechowska, M. Szpot, M. Staszel, S. Almeida, H. Pereira, L. Brandão Alves, R. Miranda, L. Ribeiro, F. Costa, F. Morgado, P. Carmo, P. Galvao Santos, R. Bernardo, P. Adragão, G. Ferreira da Silva, M. Peres, M. Alves, M. Leal, A. Cordeiro, P. Magalhães, P. Fontes, S. Leão, A. Delgado, A. Costa, B. Marmelo, B. Rodrigues, D. Moreira, J. Santos, L. Santos, A. Terchet, D. Darabantiu, S. Mercea, V. Turcin Halka, A. Pop Moldovan, A. Gabor, B. Doka, G. Catanescu, H. Rus, L. Oboroceanu, E. Bobescu, R. Popescu, A. Dan, A. Buzea, I. Daha, G. Dan, I. Neuhoff, M. Baluta, R. Ploesteanu, N. Dumitrache, M. Vintila, A. Daraban, C. Japie, E. Badila, H. Tewelde, M. Hostiuc, S. Frunza, E. Tintea, D. Bartos, A. Ciobanu, I. Popescu, N. Toma, C. Gherghinescu, D. Cretu, N. Patrascu, C. Stoicescu, C. Udroiu, G. Bicescu, V. Vintila, D. Vinereanu, M. Cinteza, R. Rimbas, M. Grecu, A. Cozma, F. Boros, M. Ille, O. Tica, R. Tor, A. Corina, A. Jeewooth, B. Maria, C. Georgiana, C. Natalia, D. Alin, D. Dinu‐Andrei, M. Livia, R. Daniela, R. Larisa, S. Umaar, T. Tamara, M. Ioachim Popescu, D. Nistor, I. Sus, O. Coborosanu, N. Alina‐Ramona, R. Dan, L. Petrescu, G. Ionescu, I. Popescu, C. Vacarescu, E. Goanta, M. Mangea, A. Ionac, C. Mornos, D. Cozma, S. Pescariu, E. Solodovnicova, I. Soldatova, J. Shutova, L. Tjuleneva, T. Zubova, V. Uskov, D. Obukhov, G. Rusanova, I. Soldatova, N. Isakova, S. Odinsova, T. Arhipova, E. Kazakevich, E. Serdechnaya, O. Zavyalova, T. Novikova, I. Riabaia, S. Zhigalov, E. Drozdova, I. Luchkina, Y. Monogarova, D. Hegya, L. Rodionova, L. Rodionova, V. Nevzorova, I. Soldatova, O. Lusanova, A. Arandjelovic, D. Toncev, L. Vukmirovic, M. Radisavljevic, M. Milanov, N. Sekularac, M. Zdravkovic, S. Hinic, S. Dimkovic, T. Acimovic, J. Saric, S. Radovanovic, A. Kocijancic, B. Obrenovic‐Kircanski, D. Kalimanovska Ostric, D. Simic, I. Jovanovic, I. Petrovic, M. Polovina, M. Vukicevic, M. Tomasevic, N. Mujovic, N. Radivojevic, O. Petrovic, S. Aleksandric, V. Kovacevic, Z. Mijatovic, B. Ivanovic, M. Tesic, T. Potpara, A. Ristic, B. Vujisic‐Tesic, M. Nedeljkovic, A. Karadzic, A. Uscumlic, M. Prodanovic, M. Zlatar, M. Asanin, B. Bisenic, V. Vasic, Z. Popovic, D. Djikic, M. Sipic, V. Peric, B. Dejanovic, N. Milosevic, S. Backovic, A. Stevanovic, A. Andric, B. Pencic, M. Pavlovic‐Kleut, V. Celic, M. Pavlovic, M. Petrovic, M. Vuleta, N. Petrovic, S. Simovic, Z. Savovic, S. Milanov, G. Davidovic, V. Iric‐Cupic, D. Djordjevic, M. Damjanovic, S. Zdravkovic, V. Topic, D. Stanojevic, M. Randjelovic, R. Jankovic‐Tomasevic, V. Atanaskovic, S. Antic, M. Pavlovic, D. Simonovic, M. Stojanovic, S. Stojanovic, V. Mitic, V. Ilic, D. Petrovic, M. Deljanin Ilic, S. Ilic, V. Stoickov, S. Markovic, A. Mijatovic, D. Tanasic, D. Petrovic, G. Radakovic, J. Peranovic, M. Pavlovic, N. Panic‐Jelic, O. Vujadinovic, P. Pajic, S. Bekic, S. Kovacevic, A. García Fernandez, A. Perez Cabeza, M. Anguita, L. Tercedor Sanchez, E. Mau, J. Loayssa, M. Ayarra, M. Carpintero, I. Roldán Rabadan, M. Leal, M. Gil Ortega, A. Tello Montoliu, E. Orenes Piñero, S. Manzano Fernández, F. Marín, A. Romero Aniorte, A. Veliz Martínez, M. Quintana Giner, G. Ballesteros, M. Palacio, O. Alcalde, I. García‐Bolao, V. Bertomeu Gonzalez, F. Otero‐Raviña, J. García Seara, J. Gonzalez Juanatey, N. Dayal, P. Maziarski, P. Gentil‐Baron, D. Shah, M. Koç, E. Onrat, I. E. Dural, K. Yilmaz, B. Özin, S. Tan Kurklu, Y. Atmaca, U. Canpolat, L. Tokgozoglu, A. K. Dolu, B. Demirtas, D. Sahin, O. Ozcan Celebi, E. Diker, G. Gagirci, U.O. Turk, H. Ari, N. Polat, N. Toprak, M. Sucu, O. Akin Serdar, A. Taha Alper, A. Kepez, Y. Yuksel, A. Uzunselvi, S. Yuksel, M. Sahin, O. Kayapinar, T. Ozcan, H. Kaya, M. B. Yilmaz, M. Kutlu, M. Demir, C. Gibbs, S. Kaminskiene, M. Bryce, A. Skinner, G. Belcher, J. Hunt, L. Stancombe, B. Holbrook, C. Peters, S. Tettersell, A. Shantsila, D. Lane, K. Senoo, M. Proietti, K. Russell, P. Domingos, S. Hussain, J. Partridge, R. Haynes, S. Bahadur, R. Brown, S. McMahon, G. Y H Lip, J. McDonald, K. Balachandran, R. Singh, S. Garg, H. Desai, K. Davies, W. Goddard, G. Galasko, I. Rahman, Y. Chua, O. Payne, S. Preston, O. Brennan, L. Pedley, C. Whiteside, C. Dickinson, J. Brown, K. Jones, L. Benham, R. Brady, L. Buchanan, A. Ashton, H. Crowther, H. Fairlamb, S. Thornthwaite, C. Relph, A. McSkeane, U. Poultney, N. Kelsall, P. Rice, T. Wilson, M. Wrigley, R. Kaba, T. Patel, E. Young, J. Law, C. Runnett, H. Thomas, H. McKie, J. Fuller, S. Pick, A. Sharp, A. Hunt, K. Thorpe, C. Hardman, E. Cusack, L. Adams, M. Hough, S. Keenan, A. Bowring, J. Watts, J. Zaman, K. Goffin, H. Nutt, Y. Beerachee, J. Featherstone, C. Mills, J. Pearson, L. Stephenson, S. Grant, A. Wilson, C. Hawksworth, I. Alam, M. Robinson, S. Ryan, R. Egdell, E. Gibson, M. Holland, D. Leonard, B. Mishra, S. Ahmad, H. Randall, J. Hill, L. Reid, M. George, S. McKinley, L. Brockway, W. Milligan, J. Sobolewska, J. Muir, L. Tuckis, L. Winstanley, P. Jacob, S. Kaye, L. Morby, A. Jan, T. Sewell, C. Boos, B. Wadams, C. Cope, P. Jefferey, N. Andrews, A. Getty, A. Suttling, C. Turner, K. Hudson, R. Austin, S. Howe, R. Iqbal, N. Gandhi, K. Brophy, P. Mirza, E. Willard, S. Collins, N. Ndlovu, E. Subkovas, V. Karthikeyan, L. Waggett, A. Wood, A. Bolger, J. Stockport, L. Evans, E. Harman, J. Starling, L. Williams, V. Saul, M. Sinha, L. Bell, S. Tudgay, S. Kemp, J. Brown, L. Frost, T. Ingram, A. Loughlin, C. Adams, M. Adams, F. Hurford, C. Owen, C. Miller, D. Donaldson, H. Tivenan, H. Button, A. Nasser, O. Jhagra, B. Stidolph, C. Brown, C. Livingstone, M. Duffy, P. Madgwick, P. Roberts, E. Greenwood, L. Fletcher, M. Beveridge, S. Earles, D. McKenzie, D. Beacock, M. Dayer, M. Seddon, D. Greenwell, F. Luxton, F. Venn, H. Mills, J. Rewbury, K. James, K. Roberts, L. Tonks, D. Felmeden, W. Taggu, A. Summerhayes, D. Hughes, J. Sutton, L. Felmeden, M. Khan, E. Walker, L. Norris, L. O’Donohoe, A. Mozid, H. Dymond, H. Lloyd‐Jones, G. Saunders, D. Simmons, D. Coles, D. Cotterill, S. Beech, S. Kidd, B. Wrigley, S. Petkar, A. Smallwood, R. Jones, E. Radford, S. Milgate, S. Metherell, V. Cottam, C. Buckley, A. Broadley, D. Wood, J. Allison, K. Rennie, L. Balian, L. Howard, L. Pippard, S. Board, T. Pitt‐Kerby

**Affiliations:** ^1^ Liverpool Centre for Cardiovascular Science, University of Liverpool and Liverpool Heart & Chest Hospital Liverpool UK; ^2^ School of Medicine University of Belgrade Belgrade Serbia; ^3^ Intensive Arrhythmia Care Cardiology Clinic, Clinical Center of Serbia Belgrade Serbia; ^4^ Department of Medical Science and Cardiology Uppsala University Uppsala Sweden; ^5^ Cardiology Division Department of Biomedical, Metabolic and Neural Sciences University of Modena and Reggio Emilia Policlinico di Modena Modena Italy; ^6^ Department of Cardiology Hospital Universitario Virgen de la Arrixaca, IMIB‐Arrixaca, University of Murcia, CIBERCV Murcia Spain; ^7^ Service de Cardiologie Centre Hospitalier Universitaire Trousseau Tours France; ^8^ Aalborg Thrombosis Research Unit, Department of Clinical Medicine Aalborg University Aalborg Denmark

**Keywords:** atrial fibrillation, chronic kidney disease, death, kidney failure, major bleeding, outcome, thromboembolism

## Abstract

**Background:**

Atrial fibrillation (AF) and renal impairment share a bidirectional relationship with important pathophysiological interactions. We evaluated the impact of renal impairment in a contemporary cohort of patients with AF.

**Methods:**

We utilised the ESC‐EHRA EORP‐AF Long‐Term General Registry. Outcomes were analysed according to renal function by CKD‐EPI equation. The primary endpoint was a composite of thromboembolism, major bleeding, acute coronary syndrome and all‐cause death. Secondary endpoints were each of these separately including ischaemic stroke, haemorrhagic event, intracranial haemorrhage, cardiovascular death and hospital admission.

**Results:**

A total of 9306 patients were included. The distribution of patients with no, mild, moderate and severe renal impairment at baseline were 16.9%, 49.3%, 30% and 3.8%, respectively. AF patients with impaired renal function were older, more likely to be females, had worse cardiac imaging parameters and multiple comorbidities. Among patients with an indication for anticoagulation, prescription of these agents was reduced in those with severe renal impairment, *p* < .001. Over 24 months, impaired renal function was associated with significantly greater incidence of the primary composite outcome and all secondary outcomes. Multivariable Cox regression analysis demonstrated an inverse relationship between eGFR and the primary outcome (HR 1.07 [95% CI, 1.01–1.14] per 10 ml/min/1.73 m^2^ decrease), that was most notable in patients with eGFR <30 ml/min/1.73 m^2^ (HR 2.21 [95% CI, 1.23–3.99] compared to eGFR ≥90 ml/min/1.73 m^2^).

**Conclusion:**

A significant proportion of patients with AF suffer from concomitant renal impairment which impacts their overall management. Furthermore, renal impairment is an independent predictor of major adverse events including thromboembolism, major bleeding, acute coronary syndrome and all‐cause death in patients with AF.

## 1 INTRODUCTION

Atrial fibrillation (AF) is the most common sustained cardiac arrhythmia and it is associated with poor outcomes. Due to the systemic nature of AF, it frequently has pathophysiological interactions with other associated comorbidities. An example is chronic kidney disease (CKD), which is defined as abnormalities involving the kidney structure or function that lasts for more than 3 months with health implications.^1^ The prevalence of both AF and renal impairment increases with age and pre‐existing comorbidities.^2^, ^3^ Moreover, there is a bidirectional relationship between these conditions such that renal impairment increases the risk of incident AF while the presence of AF promotes the development and progression of renal impairment.^4^

From a clinical perspective, there are many crucial aspects to consider when treating patients with AF and renal impairment. Most importantly, patients with AF suffer from an increased risk of all‐cause death and stroke, and these risks are amplified in those with co‐existing renal impairment.^5^ Preventive strategies include the use of anticoagulation therapy such as warfarin, which has been shown to be influenced by renal impairment and consequently linked to poor outcomes.^6^ Alternatively, non‐vitamin K antagonist oral anticoagulants (NOACs) may be used but these agents have varying degrees of renal excretion. Hence, the presence of renal impairment has a significant influence on the choice of anticoagulation in patients with AF.

Presently, the impact of renal function on long‐term outcomes in AF remains ill‐defined. The objectives of this study were to evaluate the impact of renal impairment in a contemporary cohort of AF patients across Europe by utilizing the EURObservational Research Programme in AF (EORP‐AF) General Long‐Term Registry.

## 2 METHODS

The EORP‐AF General Long‐Term Registry is a prospective, observational, large‐scale multicentre registry from 250 centres in 27 participating European countries. A detailed description of the design and baseline characteristics have previously been provided.^7^ In brief, patients with AF who presented to inpatient and outpatient cardiology services were enrolled between October 2013 and September 2016. All patients were equal or older than 18 years and had documented AF on prior electrocardiographic evaluation within 12 months of enrolment. Institutional review board approval of the study protocol was obtained for every participating organisation, informed consent was obtained from patients and the study was performed in accordance to the European Union Note for Guidance on Good Clinical Practice CPMP/ECH/135/95 and the Declaration of Helsinki. Reporting of the study conforms to broad EQUATOR guidelines.^8^

### 2.1 Data collection and definition

Data on demographics, comorbidities and management strategies were collected with prospectively designed data collection forms. Baseline serum creatinine, sex, age and ethnicity were determined at enrolment for calculation of estimated glomerular filtration rate (eGFR) using the Chronic Kidney Disease‐Epidemiology Collaboration (CKD‐EPI) equation. Patients with missing data were excluded. Based on eGFR, patients were categorised into four groups: no renal impairment (eGFR ≥90 ml/min/1.73 m^2^), mild renal impairment (eGFR 60–89 ml/min/1.73 m^2^), moderate renal impairment (eGFR 30–59 ml/min/1.73 m^2^) and severe renal impairment (eGFR <30 ml/min/1.73 m^2^). AF classification was determined according to the European Society of Cardiology recommendations.^9^ Severity of AF‐related symptoms was ascertained using the European Heart Rhythm Association (EHRA) classification.

### 2.2 Study outcomes

The primary endpoint was a composite of thromboembolism (TE), major bleeding, acute coronary syndrome (ACS) and all‐cause death. Secondary endpoints were TE, ischaemic stroke, ACS, haemorrhagic event, major bleeding, intracranial haemorrhage, all‐cause death, cardiovascular death and hospital admission. The study outcomes were recorded over a 2‐year follow‐up period. All outcomes were assessed and reported by investigators.

### 2.3 Statistical analyses

Continuous variables were tested for normality with Kolmogorov–Smirnov test. Parametric variables were described with mean and standard deviation, and were compared using *t*‐test. Non‐parametric variables were described with median and interquartile range (IQR), and tested for differences using Kruskal Wallis test, which evaluates for differences between all the groups. Categorical variables were described with count and percentage, and tested for differences using chi‐squared test or Fisher's exact test (if expected cell count was less than five). Plots of Kaplan–Meier curves for study endpoints in relation to eGFR categories were performed and survival distributions compared using log‐rank test. Cox proportional hazards model was used to establish the relationship between renal function and the risk of major adverse events in AF. To adjust for possible confounders, all variables that were significantly different between the groups at baseline with a level of significance of *p* 10 were included in the univariate analysis. Subsequently, risk factors associated with the composite outcome with *p* value below .10 were incorporated into a forward multivariate proportional hazards model. The model was repeated with the inclusion of eGFR as either a categorical or continuous covariate. A two‐sided *p* value of <.05 was considered statistically significant. Analyses were performed using SPSS software version 24 (IBM Corp).

## 3 RESULTS

A total of 9306 patients with AF were included in this analysis, corresponding to 83.9% of the original cohort of 11,096 patients. The remaining patients were excluded due to missing data for calculation of eGFR. Median age of patients was 68.8 (IQR 62.0–77.0) years with 3841 (41.3%) females. The distribution of patients with no, mild, moderate and severe renal impairment were 1573 (16.9%), 4586 (49.3%), 2790 (30.0%) and 357 (3.8%), respectively. A total of 5531 (59.4%) patients were enrolled during hospitalisation: 927 (58.9%) patients with no renal impairment, 2621 (57.2%) with mild renal impairment, 1723 (61.8%) with moderate renal impairment and 260 (72.8%) with severe renal impairment (*p* for trend <.001).

### 3.1 Baseline characteristics

Baseline patient characteristics based on renal function are summarised in Table 1. Median eGFR in the no, mild, moderate and severe renal impairment groups were 96.8 (IQR 93.0–102.6) ml/min/1.73 m^2^, 74.7 (IQR 67.6–82.6) ml/min/1.73 m^2^, 49.2 (IQR 41.6–55.2) ml/min/1.73 m^2^ and 23.7 (IQR 18.4–27.7) ml/min/1.73 m^2^, respectively. AF patients with impaired renal function were older and more likely to be females with larger left atrial diameter, lower left ventricular function and greater proportion of left ventricular hypertrophy. Furthermore, they had a greater prevalence of permanent AF and comorbidities including cardiomyopathy, chronic obstructive pulmonary disease, coronary artery disease, diabetes mellitus, heart failure, hypertension, peripheral vascular disease, prior haemorrhagic event, prior TE, hypothyroidism and valvular heart disease. This is reflected by a graded increase in CHA_2_DS_2_‐VASc and HAS‐BLED scores among patients with impaired renal function.

**TABLE 1:** Baseline patient characteristics

Baseline characteristics	Total (*n* = 9306)	eGFR ≥90 (*n* = 1573; 16.9%)	eGFR 60–89 (*n* = 4586; 49.3%)	eGFR 30–59 (*n* = 2790; 30.0%)	eGFR <30 (*n* = 357; 3.8%)	*p* value^b^
Age (years), median (IQR)	68.8 (62.0–77.0)	59.0 (50.0–65.0)	69.0 (63.0–76.0)	75.0 (69.0–81.0)	78.0 (72.0–83.0)	<.001
Female sex, *n* (%)	3841 (41.3)	484 (30.8)	1772 (38.6)	1387 (49.7)	198 (55.5)	<.001
Body mass index (kg/m^2^), median (IQR)	27.5 (24.7–31.1)	27.4 (24.6–31.2)	27.5 (24.8–30.9)	27.6 (24.7–31.2)	27.3 (24.5–31.1)	<.001
eGFR (ml/min/1.73 m^2^), median (IQR)	69.7 (54.3–84.9)	96.8 (93.0–102.6)	74.7 (67.6–82.6)	49.2 (41.6–55.2)	23.7 (18.4–27.7)	<.001
Left atrial diameter (mm), median (IQR)	45.0 (40.0–50.0)	43.0 (38.0–48.0)	45.0 (40.0–50.0)	46.0 (41.0–51.0)	47.0 (42.5–51.5)	<.001
LV ejection fraction (%), median (IQR)	55.0 (45.0–61.0)	57.0 (50.0–62.0)	55.0 (46.0–62.0)	53.0 (41.0–60.0)	50.0 (35.0–58.0)	<.001
Left ventricular hypertrophy, *n* (%)	2107 (27.0)	253 (19.2)	996 (26.1)	750 (31.8)	108 (35.8)	<.001
AF type, *n* (%)						
First‐detected	1612 (17.6)	341 (22.0)	818 (18.2)	404 (14.7)	49 (14.0)	<.001
Paroxysmal	2467 (27.0)	516 (33.2)	1250 (27.8)	613 (22.3)	88 (25.1)	
Persistent	1908 (20.9)	348 (22.4)	995 (22.1)	526 (19.1)	39 (11.1)	
Long‐standing persistent	392 (4.3)	75 (4.8)	195 (4.3)	113 (4.1)	9 (2.6)	
Permanent	2768 (30.3)	273 (17.6)	1236 (27.5)	1094 (39.8)	165 (47.1)	
Alcohol consumption ≥2 units/day, *n* (%)	638 (7.5)	157 (10.7)	341 (8.1)	133 (5.3)	7 (2.2)	<.001
Current smoker, *n* (%)	893 (10.1)	291 (19.2)	435 (9.9)	148 (5.6)	19 (5.8)	<.001
No regular exercise, *n* (%)	3574 (43.2)	460 (32.5)	1583 (38.8)	1321 (53.6)	210 (66.5)	<.001
Comorbidities, *n* (%)						
Cardiomyopathy^a^	1475 (16.0)	213 (13.7)	642 (14.1)	518 (18.8)	102 (29.0)	<.001
Congenital heart disease	112 (1.2)	25 (1.6)	55 (1.2)	28 (1.0)	4 (1.1)	.402
Chronic obstructive pulmonary disease	845 (9.1)	105 (6.7)	369 (8.1)	317 (11.4)	54 (15.2)	<.001
Coronary artery disease	2604 (29.8)	270 (18.1)	1228 (28.4)	942 (36.4)	164 (50.2)	<.001
Diabetes mellitus	2170 (23.4)	260 (16.6)	914 (20.0)	834 (30.1)	162 (45.8)	<.001
Heart failure	3797 (41.1)	412 (26.4)	1612 (35.4)	1544 (55.8)	229 (64.5)	<.001
Hypertension	5683 (61.6)	739 (47.6)	2754 (60.5)	1923 (69.4)	267 (75.6)	<.001
Liver disease	278 (3.0)	47 (3.0)	115 (2.5)	101 (3.6)	15 (4.2)	.026
Malignancy	192 (2.1)	28 (1.8)	81 (1.8)	66 (2.4)	17 (4.8)	.001
Peripheral vascular disease	776 (8.5)	67 (4.3)	338 (7.5)	315 (11.6)	56 (16.2)	<.001
Previous haemorrhagic event	500 (5.4)	69 (4.4)	194 (4.3)	201 (7.3)	36 (10.2)	<.001
Previous thromboembolic event	1109 (12.0)	102 (6.5)	546 (12.0)	399 (14.5)	62 (17.7)	<.001
Previous ischaemic stroke	600 (6.5)	52 (3.3)	292 (6.4)	216 (7.8)	40 (11.4)	<.001
Previous transient ischaemic attack	288 (3.1)	26 (1.7)	151 (3.3)	100 (3.6)	11 (3.1)	.003
Sleep apnoea	406 (4.5)	79 (5.2)	187 (4.2)	124 (4.6)	16 (4.7)	.172
Hyperthyroidism	161 (1.8)	37 (2.4)	81 (1.8)	40 (1.5)	3 (0.9)	.081
Hypothyroidism	517 (5.7)	52 (3.4)	222 (4.9)	212 (7.8)	31 (8.8)	<.001
Valvular heart disease	4521 (49.6)	534 (34.6)	2157 (47.9)	1598 (58.7)	232 (66.9)	<.001
Prior cardioversion for AF, *n* (%)						
Electrical cardioversion	1419 (16.6)	302 (20.6)	695 (16.4)	389 (15.3)	33 (10.2)	<.001
Pharmacological cardioversion	1710 (20.0)	356 (24.3)	846 (20.0)	449 (17.6)	59 (18.2)	.010
Prior catheter AF ablation, *n* (%)	428 (5.8)	99 (8.3)	211 (5.8)	109 (4.8)	9 (3.2)	<.001
Risk scores						
CHA_2_DS_2_‐VASc score, median (IQR)	3 (2–4)	2 (1–3)	3 (2–4)	4 (3–5)	5 (4–6)	<.001
HAS‐BLED score, median (IQR)	2 (1–2)	1 (0–1)	1 (1–2)	2 (1–3)	3 (2–3)	<.001

Abbreviations: AF, atrial fibrillation; eGFR, estimated glomerular filtration rate; IQR, interquartile range; LV, left ventricle.

^a^ Includes all forms of cardiomyopathy as determined by the responsible physician.

^b^ Categorical variables are evaluated by *p* value for trend.

Atrial fibrillation was reported to be the primary diagnosis at baseline admission or consultation in most patients (Table S1 **)**. Nonetheless, the frequency of other primary diagnoses including non‐cardiovascular conditions was increased with impaired renal function. Additionally, these patients were significantly more likely to have severe symptoms attributable to AF. Recent diagnostics and baseline interventions performed according to renal function are provided in Table S2.

### 3.2 Management strategies

**FIGURE 1:**
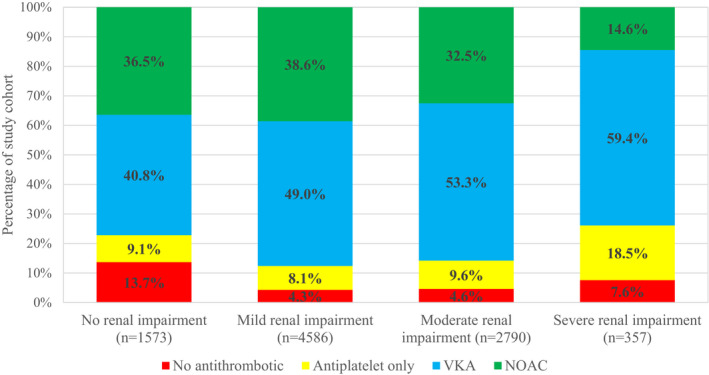
Antithrombotic regime in patients with a valid indication at baseline according to renal function. VKA, vitamin K antagonist; NOAC, non‐vitamin K antagonist oral anticoagulant; OAC, oral anticoagulant

Overall, there was a good rate of prescription of oral anticoagulants (*n* = 8061 [86.7%]) in this patient cohort (Table 2). There were 1066 (11.5%) patients on reduced doses of NOAC therapy. When the analysis was limited to the subgroup of patients in whom anticoagulation was indicated (CHA_2_DS_2_‐VASc ≥2 [male] or ≥3 [female]; *n* = 6998), prescription of these agents was significantly reduced in those with severe renal impairment (78.6%). Furthermore, there was an increased preference for vitamin K antagonist (VKA) over NOACs with impaired renal function (Figure 1).

**TABLE 2:** Baseline medication use

Medication use	Total (*n* = 9306)	eGFR ≥90 (*n* = 1573; 16.9%)	eGFR 60–89 (*n* = 4586; 49.3%)	eGFR 30–59 (*n* = 2790; 30.0%)	eGFR <30 (*n* = 357; 3.8%)	*p* value (trend)
Anti‐thrombotic, *n* (%)	8731 (93.9)	1356 (86.3)	4388 (95.7)	2657 (95.4)	330 (92.4)	<.001
Any antiplatelet	1994 (21.4)	269 (17.1)	921 (20.1)	678 (24.3)	126 (35.3)	<.001
Acetylsalicylic acid	1793 (19.3)	255 (16.2)	828 (18.1)	595 (21.4)	115 (32.2)	<.001
Clopidogrel	648 (7.0)	70 (4.5)	297 (6.5)	238 (8.5)	43 (12.0)	<.001
Oral anticoagulants, *n* (%)	8061 (86.7)	1235 (78.6)	4092 (89.2)	2454 (88.1)	280 (78.4)	<.001
Vitamin K antagonist	4584 (49.3)	641 (40.8)	2247 (49.0)	1484 (53.3)	212 (59.4)	<.001
Any NOAC	3299 (35.5)	573 (36.5)	1768 (38.6)	906 (32.5)	52 (14.6)	<.001
Apixaban	936 (10.1)	139 (8.8)	489 (10.7)	285 (10.2)	23 (6.4)	<.001
Dabigatran	750 (8.1)	140 (8.9)	398 (8.7)	205 (7.4)	7 (2.0)	<.001
Edoxaban	38 (0.4)	5 (0.3)	29 (0.6)	4 (0.1)	0 (0.0)	<.001
Rivaroxaban	1575 (16.9)	289 (18.4)	852 (18.6)	412 (14.8)	22 (6.2)	<.001
Anti‐arrhythmics, *n* (%)	2723 (29.3)	566 (36.1)	1340 (29.3)	727 (26.1)	90 (25.2)	<.001
Amiodarone	1745 (18.8)	276 (17.6)	820 (17.9)	567 (20.4)	82 (23.0)	<.001
Flecainide	324 (3.5)	101 (6.4)	173 (3.8)	45 (1.6)	5 (1.4)	<.001
Propafenone	361 (3.9)	119 (7.6)	181 (4.0)	60 (2.2)	1 (0.3)	<.001
Sotalol	271 (2.9)	66 (4.2)	154 (3.4)	49 (1.8)	2 (0.6)	<.001
Other treatments, *n* (%)						
ACE inhibitors	3945 (42.5)	587 (37.4)	1973 (43.1)	1258 (45.3)	127 (35.6)	<.001
Aldosterone blockers	1793 (19.3)	192 (12.3)	814 (17.8)	723 (26.0)	64 (17.9)	<.001
Angiotensin receptor blocker	1725 (18.6)	195 (12.4)	883 (19.3)	595 (21.4)	52 (14.6)	<.001
Beta‐blockers	6390 (68.9)	1044 (66.7)	3113 (68.0)	1981 (71.2)	252 (70.8)	.005
Dihydropyridine CCB	1588 (17.1)	224 (14.3)	771 (16.9)	507 (18.2)	86 (24.1)	<.001
Digoxin	1352 (14.6)	176 (11.2)	628 (13.7)	489 (17.6)	59 (16.5)	<.001
Diuretics	4857 (52.4)	525 (33.5)	2210 (48.3)	1850 (66.5)	272 (76.4)	<.001
Insulin	516 (5.6)	40 (2.6)	169 (3.7)	237 (8.5)	70 (19.6)	<.001
Oral antidiabetics	1460 (15.7)	190 (12.1)	650 (14.2)	535 (19.2)	85 (23.8)	<.001
Statins	3975 (42.9)	478 (30.5)	1997 (43.7)	1328 (47.7)	172 (48.2)	<.001

Abbreviations: ACE, angiotensin converting enzyme; CCB, calcium‐channel blocker; eGFR, estimated glomerular filtration rate; NOAC, non‐vitamin K antagonist oral anticoagulant.

In terms of rhythm control strategies, patients with impaired renal function were less likely to be prescribed anti‐arrhythmic medications at baseline. Moreover, although few (20.6%) patients in this cohort received rhythm control interventions during the follow‐up period, this was particularly evident in those with worse renal function (11.5% in patient with severe renal impairment vs. 26.1% in patients with no renal impairment), *p* < .001.

### 3.3 Major adverse events

During a median follow‐up of 24 months, there were a total of 727 (8.6%) recorded events for the composite outcome of TE, major bleeding, ACS and all‐cause death (Table 3). In this regard, patients with worse renal function had significantly more adverse events (*p* < .001) with rates of 4.9%, 7.5%, 11.4% and 17.6% in the no, mild, moderate and severe renal impairment groups, respectively. A total of 842 (9.0%) patients were lost to follow‐up for the primary endpoint. Worse renal function was associated with significantly higher incidences of any TE (*p* = .043), ischaemic stroke (*p* = .034), ACS (*p* < .001), any haemorrhagic event (*p* < .001), major bleeding (*p* = .002), intracranial haemorrhage (*p* = .010), all‐cause death (*p* < .001), cardiovascular death (*p* < .001) and hospital admissions (*p* < .001).

**TABLE 3:** Major adverse events at 2‐years according to renal function

Major adverse events, *n* (%)	Total (*n* = 9306)	eGFR ≥90 (*n* = 1573; 16.9%)	eGFR 60–89 (*n* = 4586; 49.3%)	eGFR 30–59 (*n* = 2790; 30.0%)	eGFR <30 (*n* = 357; 3.8%)	*p* value (trend)
Composite of thromboembolism, major bleeding, acute coronary syndrome and all‐cause death	727 (8.6)	71 (4.9)	313 (7.5)	289 (11.4)	54 (17.6)	<.001
Any thromboembolism	173 (2.1)	22 (1.6)	77 (1.9)	66 (2.7)	8 (2.7)	.043
Ischaemic stroke	93 (1.1)	8 (0.6)	45 (1.1)	34 (1.4)	6 (2.0)	.034
Acute coronary syndrome	208 (2.5)	23 (1.6)	101 (2.4)	71 (2.8)	13 (4.2)	<.001
Haemorrhagic event	306 (3.6)	34 (2.4)	140 (3.3)	111 (4.4)	21 (6.8)	<.001
Major bleeding	83 (1.0)	4 (0.3)	40 (1.0)	34 (1.3)	5 (1.6)	.002
Intracranial haemorrhage	28 (0.3)	0 (0.0)	13 (0.3)	14 (0.6)	1 (0.3)	.010
All‐cause death	850 (9.1)	68 (4.3)	301 (6.6)	373 (13.4)	108 (30.3)	<.001
Cardiovascular death	341 (3.8)	20 (1.3)	118 (2.6)	153 (5.8)	50 (15.5)	<.001
Any admission	2046 (25.7)	327 (23.6)	997 (24.8)	648 (28.1)	74 (31.1)	<.001
AF‐related admission	1237 (14.7)	256 (17.9)	606 (14.5)	345 (13.7)	30 (9.8)	<.001

Abbreviations: AF, atrial fibrillation, eGFR, estimated glomerular filtration rate.

**FIGURE 2:**
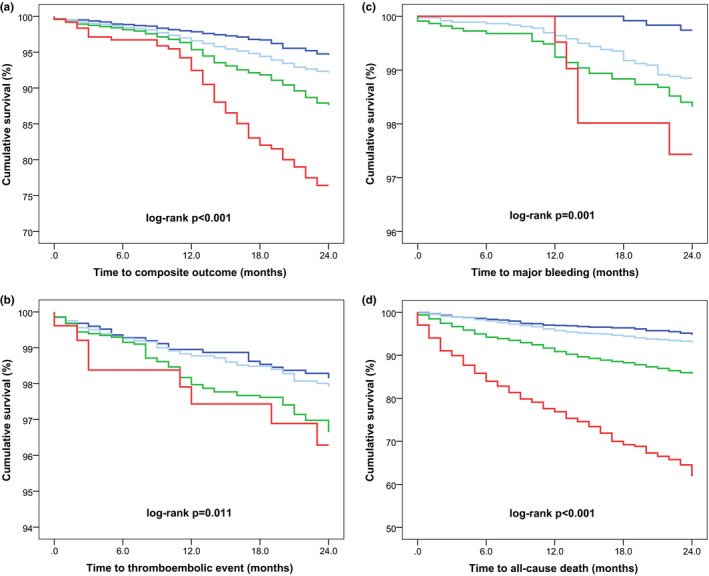
Kaplan–Meier curves for the composite outcome of thromboembolism, major bleeding, acute coronary syndrome and all‐cause death (A), thromboembolism (B), major bleeding (C) and all‐cause death (D) according to renal function. No renal impairment is represented by dark blue, mild renal impairment by light blue, moderate renal impairment by green and severe renal impairment by red

In comparison to patients with no renal impairment, those with mild, moderate and severe renal impairment had crude hazard ratios (HRs) for the composite outcome of 1.50 (95% CI, 1.16–1.96), 2.40 (95% CI, 1.84–3.14) and 4.87 (95% CI, 3.38–7.00), respectively (Table 4). A similar trend was observed for the risk of any TE, ischaemic stroke, major bleeding, ACS, all‐cause death and cardiovascular death. Kaplan–Meier survival analyses demonstrated that worse renal function in AF was linked to significantly increased risk for time to the composite outcome of TE, major bleeding, ACS and all‐cause death (log‐rank *p* < .001), any TE (log‐rank *p* = .007), major bleeding (log‐rank *p* < .001) and all‐cause death (log‐rank *p* < .001) (Figure 2).

**TABLE 4:** Effects of renal impairment on 2‐year outcomes in atrial fibrillation

2‐Year outcomes	eGFR ≥90 (*n* = 1573; 16.9%)	eGFR 60–89 (*n* = 4586; 49.3%)	eGFR 30–59 (*n* = 2790; 30.0%)	eGFR <30 (*n* = 357; 3.8%)
	Unadjusted HR (95% CI)			
Composite of thromboembolism, major bleeding, acute coronary syndrome and all‐cause death	Ref.	1.50 (1.16–1.96)	2.40 (1.84–3.14)	4.87 (3.38–7.00)
Any thromboembolism	Ref.	1.17 (0.73–1.88)	1.84 (1.13–2.98)	2.30 (1.02–5.17)
Ischaemic stroke	Ref.	1.91 (0.90–4.05)	2.65 (1.22–5.71)	4.74 (1.65–13.67)
Major bleeding	Ref.	3.51 (1.26–9.82)	5.20 (1.84–14.68)	8.53 (2.29–31.76)
Acute coronary syndrome	Ref.	1.60 (1.012.53)	1.97 (1.22–3.19)	3.79 (1.91–7.52)
All‐cause death	Ref.	1.43 (1.09–1.87)	3.09 (2.37–4.03)	8.94 (6.54–12.22)
Cardiovascular death	Ref.	1.94 (1.20–3.12)	4.54 (2.85–7.25)	14.09 (8.35–23.78)

Abbreviations: CI, confidence interval; eGFR, estimated glomerular filtration rate; HR, hazard ratio.

### 3.4 Multivariable analyses

**FIGURE 3:**
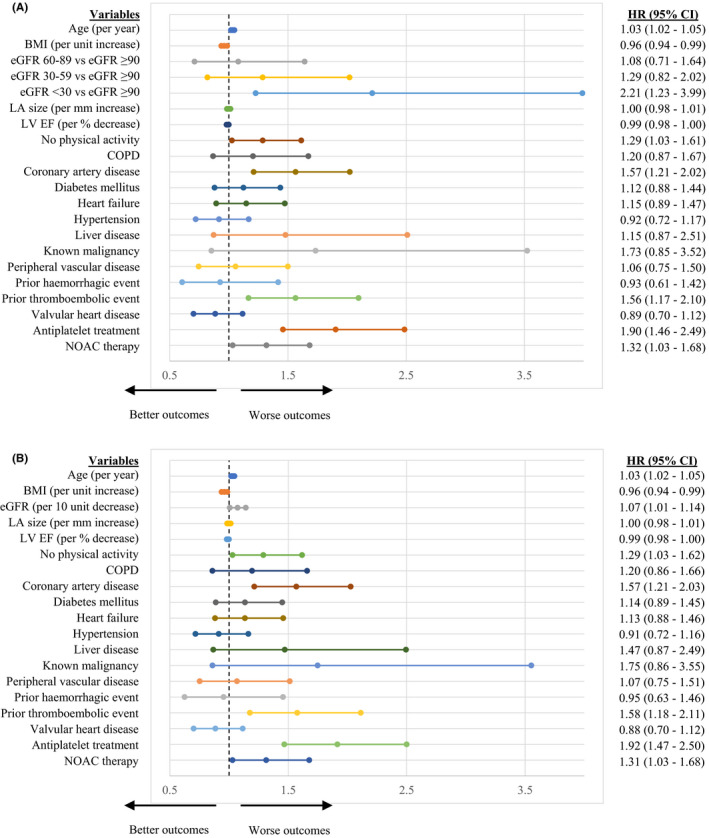
Multivariable Cox regression analysis for independent predictors of the composite outcome of thromboembolism, major bleeding, acute coronary syndrome and all‐cause death with eGFR as a categorical (A) and continuous (B) covariate. Dashes represent hazard ratio and line edges represent limits of 95% confidence intervals. BMI, body mass index, CI, confidence intervals; COPD, chronic obstructive pulmonary disease; eGFR, estimated glomerular filtration rate; HR, hazard ratio; LA, left atrial; LV EF, left ventricular ejection fraction; NOAC, non‐vitamin K antagonist oral anticoagulant

The relationship between renal function and risk of major adverse events in AF was determined after adjustment for other important risk factors identified on univariate analyses: age, body mass index, left atrial size, left ventricular function, exercise frequency, chronic obstructive pulmonary disease, coronary artery disease, diabetes mellitus, heart failure, hypertension, liver disease, known malignancy peripheral vascular disease, prior haemorrhagic event, prior TE, valvular heart disease, antiplatelet treatment and NOAC therapy (Table S3). Multivariable Cox regression analysis using eGFR as a categorical variable found that severe renal impairment was an independent predictor of the composite outcome of TE, major bleeding, ACS and all‐cause death with an adjusted HR of 2.21 (95% CI, 1.23–3.99) [Figure 3]. No significant association between mild and moderate renal impairment, and adverse outcomes in AF were found. Repeat testing using eGFR as a continuous variable demonstrated that each decrease of 10 ml/min/1.73 m^2^ in renal function contributed to an adjusted HR of 1.07 (95% CI, 1.01–1.14) for the primary composite outcome.

## 4 DISCUSSION

Our main findings from this large, contemporary, prospective study across 250 centres in 27 participating European countries of patients with AF are that impaired renal function was associated with: (1) increased age, female sex, unfavourable cardiac imaging parameters and greater prevalence of other comorbidities, (2) greater CHA_2_DS_2_‐VASc and HAS‐BLED scores, (3) reduced use of rhythm control interventions and anticoagulation therapy, the latter of which was more frequently achieved using VKA over NOACs and (4) higher incidence of adverse events including composite outcome of TE, major bleeding, ACS and all‐cause death; any TE; ischaemic stroke; ACS; any haemorrhagic event; major bleeding; intracranial haemorrhage; all‐cause death; cardiovascular death and hospital admissions. In fact, worse renal function and eGFR below 30 ml/min/1.73 m^2^ remained an independent predictor for the composite outcome of TE, major bleeding, ACS and all‐cause death, after accounting for other risk factors. Furthermore, 83% of patients with AF in this cohort had evidence of reduced renal function.

### 4.1 Baseline parameters and management strategies

It was relatively unsurprising that AF patients with impaired renal function were older with greater structural and functional changes detected on cardiac imaging alongside multiple comorbidities. However, reasons for an increased proportion of females among patients with reduced renal function remain unclear. Similar findings have also been reported in other observational studies,^10^, ^11^ despite prior evidence suggesting that female sex may have a renoprotective effect secondary to oestrogen.^12^ A potential explanation may be that male patients with AF have worse outcomes in terms of increased mortality rates compared to females, although this was not supported by our study.

Though rhythm control strategies are typically indicated for symptom relief, we found that rhythm control interventions in AF were reduced among patients with impaired renal function despite having a greater burden of AF‐related symptoms. Indeed, the ORBIT‐AF registry found that the rhythm control strategy was used less frequently among patients with creatinine clearance (CrCl) of less than 60 ml/min.^13^ This may relate to the perception that these patients have a lower probability of maintaining sinus rhythm. Schmidt et al. found that patients with an eGFR below 60 ml/min had significantly increased risk of AF recurrence at 1‐month following cardioversion.^14^ A prospective study of patients with new‐onset AF post‐ACS in the GUSTO (Global Use of Strategies to Open Occluded Coronary Arteries)‐III trial found that rhythm control in those with CrCl less than 60 ml/min was associated with lower odds of sinus rhythm at discharge compared to their counterparts.^15^ Nevertheless, this was not statistically significant after adjusting for other risk factors.

Our data highlights the fact that among AF patients with a valid indication for anticoagulation, those with an eGFR below 30 ml/min/1.73 m^2^ were less likely to receive treatment. This may reflect the current literature which do not provide sufficient evidence in favour of anticoagulation in this subgroup of patients who are exposed to an increased risk of bleeding.^4^ Furthermore, there was a preference for VKA therapy over NOACs, which remain untested in this high‐risk subgroup. An in‐depth analysis of the GARFIELD‐AF (Global Anticoagulant Registry in the FIELD–Atrial Fibrillation) registry found that moderate or worse CKD (eGFR <60 ml/min/1.73 m^2^) was strongly associated with the choice for anticoagulant therapy, with more frequent use of VKA.^16^

### 4.2 Rate of adverse events

In the SPAF (Stroke Prevention in Atrial Fibrillation) 3 trial, patients with eGFR below 60 ml/min had a twofold greater risk of ischaemic stroke or systemic embolism.^17^ In GARFIELD‐AF, even mild CKD (eGFR 60–89 ml/min/1.73 m^2^) was an independent risk factor for all‐cause mortality, after adjusting for baseline characteristics and antithrombotic use.^10^ Furthermore, moderate or severe CKD was independently associated with a higher risk of stroke or systemic embolism, major bleeding, new‐onset ACS and new or worsening heart failure. Interestingly, the impact of renal impairment was significantly greater in patients from Asia than elsewhere.^10^ However, as individual CKD status was recorded by the respective physicians, this observation may be due to misclassification bias secondary to variations in screening practices for CKD. Moreover, there may be differences in the performance of formulas used to calculate eGFR across the various ethnicities.

A retrospective cohort study showed that moderate and severe CKD were associated with an increased risk of stroke, bleeding and mortality in patients with AF.^18^ In the EORP‐AF General Pilot Registry, impaired renal function was independently associated with increased risk of stroke, transient ischaemic attack or death.^11^ Additionally, the authors highlight the prognostic implications of using different formulas for determining eGFR. The ATRIA (Anticoagulation and Risk Factors in Atrial Fibrillation) study found that eGFR below 45 ml/min/1.73 m^2^ and proteinuria were each independently associated with increased risk of TE.^19^ The latter suggests that an assessment of other markers for kidney damage should not be overlooked. In contrast, a study from the Loire Valley Atrial Fibrillation Project demonstrated that reduced renal function was not an independent predictor for ischaemic stroke or TE.^20^

Our analysis confirms that there is an inverse relationship between renal function and long‐term major adverse events including TE, major bleeding, ACS and all‐cause death among patients with AF, independent of other risk factors. Furthermore, patients with lower eGFR had more hospital admissions, though because of their greater burden of comorbidities, this was less likely to be due to AF *per se*. Unlike many of the aforementioned studies which were limited to 1‐year outcomes, we found that the impact of renal impairment on AF persisted at 2‐year follow‐up even after rigorous adjustment for possible confounders. Therefore, patients with AF and renal impairment should be considered for a more holistic and integrated care approach,^21^ which has been associated with improved clinical outcomes.^22^, ^23^

### 4.3 Prevalence of renal impairment in AF

As many as 83% of patients with AF in our cohort had reduced renal function, including 33% with moderate or severe CKD. Few patients had eGFR less than 30 ml/min/1.73 m^2^. Similar rates were reported in a retrospective cohort study of 116,506 patients with AF from an electronic record database in Israel^18^ and in other studies.^19^, ^20^

Overall, while a significant proportion of patients with AF have reduced renal function, the true prevalence of renal impairment is not well‐defined. Though patients with eGFR below 60 ml/min/1.73 m^2^ fulfil the criteria for a diagnosis of CKD, labelling those with eGFR between 60 and 89 ml/min/1.73 m^2^ as having ‘mild CKD’ and equal or more than 90 ml/min/1.73 m^2^ as ‘no CKD’ is not strictly accurate. Indeed, based on the current guidelines, the presence of a marker of kidney damage such as albuminuria, urine sediment abnormalities and structural abnormalities detected by imaging is required to diagnose CKD in patients with eGFR equal or more than 60 ml/min/1.73 m^2^.^24^, ^25^ In this regard, majority of studies in AF have relied solely on eGFR when referring to CKD. This may provide an explanation for the neutral effects of mild CKD in patients with AF. Additionally, misclassification bias within the ‘no CKD’ group may contribute to diminished effect sizes when compared to patients with moderate or worse CKD, thereby under‐estimating the true impact of renal impairment on outcomes in AF.

### 4.4 Limitations

The main limitations of our analysis were related to its observational nature and the fact that there were few patients with severe renal impairment (eGFR <30 ml/min/1.73 m^2^). Nonetheless, our findings were consistent with many other studies in this area, as discussed above. The EORP‐AF General Long‐Term Registry was based exclusively on cardiology practices and should be interpreted with caution in the wider AF population. Moreover, the association of enrolling centres with European Society of Cardiology activities may have contributed to increased anticoagulation uptake. Furthermore, residual bias may be present due to factors that were unaccounted for, and we lacked follow‐up data on renal function. Despite these limitations, our data provides a comprehensive overview and useful insights into the current practice involving AF patients with renal impairment across Europe.

## 5 CONCLUSIONS

A significant proportion of patients with AF suffer from concomitant renal impairment which is associated with greater CHA_2_DS_2_‐VASc and HAS‐BLED scores, and reduced use of rhythm control interventions and anticoagulation therapy. Furthermore, renal impairment is an independent predictor of major adverse events including TE, major bleeding, ACS and all‐cause death during long‐term follow‐up in AF. Therefore, a holistic approach that embeds clinical risk stratification should be utilised among these patients.

## CONFLICT OF INTEREST

GB: small speaker's fees from Medtronic, Boston, Biotronik, Boehringer and Bayer, outside of the submitted work. FM: receiving grants from Ferrer, and personal fees from Bayer, Pfizer/BMS. Boehringer‐Ingelheim and Astra‐Zeneca outside the submitted work. CBL: receiving grants from Medtronic, Cardiome and personal fees from Bayer, Sanofi, Boston Scientific and Merck Sharp & Dohme outside the submitted work. TSP: Consultant for Bayer and Pfizer, no fees. LF: consultant or speaker fees of small amounts for Bayer, BMS/Pfizer, Boehringer Ingelheim, Medtronic and Novartis outside of this work. GYHL: Consultant and speaker for BMS/Pfizer, Boehringer Ingelheim and Daiichi‐Sankyo. No fees are received personally. Other authors declare no conflict of interest.

## Supporting information

App S1

## Appendix

**EURObservational Research Programme Atrial Fibrillation (EORP‐AF) Long‐Term General Registry Committees and Investigators (ESC Member Countries)**

**Executive committee**: G. Boriani (Chair), G.Y.H. Lip, L. Tavazzi, A. P. Maggioni, G‐A. Dan, T. Potpara, M. Nabauer, F. Marin, Z. Kalarus, L. Fauchier.

**Steering Committee (National Coordinators)**: A. Goda, G. Mairesse, T. Shalganov, L. Antoniades, M. Taborsky, S. Riahi, P. Muda, I. García Bolao, O. Piot, M. Nabauer, K. Etsadashvili, E. Simantirakis, M. Haim, A. Azhari, J. Najafian, M. Santini, E. Mirrakhimov, K.a Kulzida, A. Erglis, L. Poposka, M. Burg, H. Crijns, Ö. Erküner, D. Atar, R. Lenarczyk, M. Martins Oliveira, D. Shah, G‐A. Dan, E. Serdechnaya, T. Potpara, E. Diker, G.Y.H. Lip, D. Lane.

**Participants: Albania** Durrës: E. Zëra, Tirana: U. Ekmekçiu, V. Paparisto, M. Tase, Tirana: H. Gjergo, J. Dragoti, A. Goda, **Belgium** Bastogne: M. Ciutea, N. Ahadi, Z. el Husseini, M. Raepers, Gilly: J. Leroy, P. Haushan, A. Jourdan, Haine Saint Paul: C. Lepiece, Hasselt: L. Desteghe, J. Vijgen, P. Koopman, G. Van Genechten, H. Heidbuchel, Kortrijk: T. Boussy, M. De Coninck, H. Van Eeckhoutte, N. Bouckaert, La Louviere: A. Friart, J. Boreux, C. Arend, Liege: P. Evrard, Liège: L. Stefan, E. Hoffer, J. Herzet, M. Massoz, Liège: C. Celentano, M. Sprynger, L. Pierard, Liège: P. Melon, Overpelt: B. Van Hauwaert, C. Kuppens, D. Faes, D. Van Lier, A. Van Dorpe, Waremme: A. Gerardy, Yvoir: O. Deceuninck, O. Xhaet, F. Dormal, E. Ballant, D. Blommaert, **Bulgaria** Pleven: D. Yakova, M. Hristov, T. Yncheva, N. Stancheva, S. Tisheva, Plovdiv: M. Tokmakova, F. Nikolov, D. Gencheva, Sofia: T. Shalganov, B. Kunev, M. Stoyanov, Sofia: D. Marchov, V. Gelev, V. Traykov, Varna: A. Kisheva, H. Tsvyatkov, R. Shtereva, S. Bakalska‐Georgieva, S. Slavcheva, Y. Yotov, **CZECH REPUBLIC** Ústí nad Labem: M. Kubíčková, **Denmark** Aalborg: A. Marni Joensen, A. Gammelmark, L. Hvilsted Rasmussen, P. Dinesen, S. Riahi, S. Krogh Venø, B. Sorensen, A. Korsgaard, K. Andersen, C. Fragtrup Hellum, Esbjerg: A. Svenningsen, O. Nyvad, P. Wiggers, Herning: O. May, A. Aarup, B. Graversen, L. Jensen, M. Andersen, M. Svejgaard, S. Vester, S. Hansen, V. Lynggaard, Madrid: M. Ciudad, Tallinn: R. Vettus, Tartu: P. Muda, **Estonia** Elche, Alicante: A. Maestre, Toledo: S. Castaño, **France** Abbeville: S. Cheggour, Abbeville: J. Poulard, V. Mouquet, S. Leparrée, Aix‐en‐Provence: J. Bouet, J. Taieb, Amiens: A. Doucy, H. Duquenne, Angers: A. Furber, J. Dupuis, J. Rautureau, Aurillac: M. Font, P. Damiano, Avignon Cedex: M. Lacrimini, Brest: J. Abalea, S. Boismal, T. Menez, J. Mansourati, Chartres: G. Range, H. Gorka, C. Laure, C. Vassalière, Creteil: N. Elbaz, N. Lellouche, K. Djouadi, Montpellier: F. Roubille, D. Dietz, J. Davy, Nimes: M. Granier, P. Winum, C. Leperchois‐Jacquey, Paris: H. Kassim, E. Marijon, J. Le Heuzey, Paris: J. Fedida, C. Maupain, C. Himbert, E. Gandjbakhch, F. Hidden‐Lucet, G. Duthoit, N. Badenco, T. Chastre, X. Waintraub, M. Oudihat, J. Lacoste, C. Stephan, Pau: H. Bader, N. Delarche, L. Giry, Pessac: D. Arnaud, C. Lopez, F. Boury, I. Brunello, M. Lefèvre, R. Mingam, M. Haissaguerre, Rennes: M. Le Bidan, D. Pavin, V. Le Moal, C. Leclercq, Saint Denis: O. Piot, T. Beitar, Saint Etienne: I. Martel, A. Schmid, N. Sadki, C. Romeyer‐Bouchard, A. Da Costa, Tours: I. Arnault, M. Boyer, C. Piat, L. Fauchier, **FYR MACEDONIA** Bitola: N. Lozance, S. Nastevska, Ohrid: A. Doneva, B. Fortomaroska Milevska, B. Sheshoski, K. Petroska, N. Taneska, N. Bakrecheski, Skopje: K. Lazarovska, S. Jovevska, V. Ristovski, A. Antovski, Skopje: E. Lazarova, I. Kotlar, J. Taleski, L. Poposka, S. Kedev, Skopje: N. Zlatanovik, Štip: S. Jordanova, T. Bajraktarova Proseva, S. Doncovska, **Georgia** Tbilisi: D. Maisuradze, A. Esakia, E. Sagirashvili, K. Lartsuliani, N. Natelashvili, N. Gumberidze, R. Gvenetadze, Tbilisi: K. Etsadashvili, N. Gotonelia, N. Kuridze, Tbilisi: G. Papiashvili, I. Menabde, **Germany** Aachen: S. Glöggler, A. Napp, C. Lebherz, H. Romero, K. Schmitz, M. Berger, M. Zink, S. Köster, J. Sachse, E. Vonderhagen, G. Soiron, K. Mischke, Bad Reichenhall: R. Reith, M. Schneider, Berlin: W. Rieker, Biberach: D. Boscher, A. Taschareck, A. Beer, Boppard: D. Oster, Brandenburg: O. Ritter, J. Adamczewski, S. Walter, Chemnitz: A. Frommhold, E. Luckner, J. Richter, M. Schellner, S. Landgraf, S. Bartholome, Chemnitz: R. Naumann, J. Schoeler, Dachau: D. Westermeier, F. William, K. Wilhelm, M. Maerkl, Detmold: R. Oekinghaus, M. Denart, M. Kriete, U. Tebbe, Ebersbach: T. Scheibner, Erlangen: M. Gruber, A. Gerlach, C. Beckendorf, L. Anneken, M. Arnold, S. Lengerer, Z. Bal, C. Uecker, H. Förtsch, S. Fechner, V. Mages, Friedberg: E. Martens, H. Methe, Göttingen: T. Schmidt, Hamburg: B. Schaeffer, B. Hoffmann, J. Moser, K. Heitmann, S. Willems, S. Willems, Hartmannsdorf: C. Klaus, I. Lange, Heidelberg: M. Durak, E. Esen, Itzehoe: F. Mibach, H. Mibach, Kassel: A. Utech, Kirchzarten: M. Gabelmann, R. Stumm, V. Ländle, Koblenz: C. Gartner, C. Goerg, N. Kaul, S. Messer, D. Burkhardt, C. Sander, R. Orthen, S. Kaes, Köln: A. Baumer, F. Dodos, Königsbrück: A. Barth, G. Schaeffer, Leisnig: J. Gaertner, J. Winkler, Leverkusen: A. Fahrig, J. Aring, I. Wenzel, Limburg: S. Steiner, A. Kliesch, E. Kratz, K. Winter, P. Schneider, Ludwigsburg: A. Haag, I. Mutscher, R. Bosch, Markkleeberg: J. Taggeselle, S. Meixner, Meissen: A. Schnabel, Meppen: A. Shamalla, H. Hötz, A. Korinth, Merzig: C. Rheinert, Moosburg: G. Mehltretter, Mühldorf: B. Schön, N. Schön, A. Starflinger, E. Englmann, Munich: G. Baytok, T. Laschinger, G. Ritscher, Munich: A. Gerth, Münster: D. Dechering, L. Eckardt, Nienburg: M. Kuhlmann, N. Proskynitopoulos, Paderborn: J. Brunn, K. Foth, Pirna: C. Axthelm, H. Hohensee, K. Eberhard, S. Turbanisch, Plauen: N. Hassler, A. Koestler, Riesa: G. Stenzel, Riesa: D. Kschiwan, M. Schwefer, S. Neiner, S. Hettwer, Rotenburg a.d. Fulda: M. Haeussler‐Schuchardt, R. Degenhardt, S. Sennhenn, S. Steiner, Starnberg: M. Brendel, Westerstede: A. Stoehr, W. Widjaja, S. Loehndorf, A. Logemann, J. Hoskamp, J. Grundt, Zorneding: M. Block, Zwiesel: R. Ulrych, A. Reithmeier, V. Panagopoulos, **Italy** Bologna: C. Martignani, D. Bernucci, E. Fantecchi, I. Diemberger, M. Ziacchi, M. Biffi, P. Cimaglia, J. Frisoni, G. Boriani, Firenze: I. Giannini, S. Boni, S. Fumagalli, S. Pupo, A. Di Chiara, P. Mirone, Modena: E. Fantecchi, G. Boriani, F. Pesce, C. Zoccali, V.L. Malavasi, **Kazakhstan** Almaty: A. Mussagaliyeva, B. Ahyt, Z. Salihova, K. Koshum‐Bayeva, **Kyrgyzstan** Bishkek: A. Kerimkulova, A. Bairamukova, E. Mirrakhimov, **Latvia** Riga: B. Lurina, R. Zuzans, S. Jegere, I. Mintale, K. Kupics, K. Jubele, A. Erglis, O. Kalejs, **Malta** Birkirkara: K. Vanhear, M. Burg, M. Cachia, E. Abela, S. Warwicker, T. Tabone, R. Xuereb, **Montenegro** Podgorica: D. Asanovic, D. Drakalovic, M. Vukmirovic, N. Pavlovic, L. Music, N. Bulatovic, A. Boskovic, the **Netherlands** Almere: H. Uiterwaal, N. Bijsterveld, Amsterdam: J. De Groot, J. Neefs, N. van den Berg, F. Piersma, A. Wilde, Delfzijl: V. Hagens, Enschede: J. Van Es, J. Van Opstal, B. Van Rennes, H. Verheij, W. Breukers, Heerenveen: G. Tjeerdsma, R. Nijmeijer, D. Wegink, R. Binnema, Hengelo: S. Said, Maastricht: Ö. Erküner, S. Philippens, W. van Doorn, H. Crijns, Rotterdam: T. Szili‐Torok, R. Bhagwandien, P. Janse, A. Muskens, s‐Hertogenbosch: M. van Eck, R. Gevers, N. van der Ven, Venlo: A. Duygun, B. Rahel, J. Meeder, **Norway** Oslo: A. Vold, C. Holst Hansen, I. Engset, D. Atar, **Poland** Bytom: B. Dyduch‐Fejklowicz, E. Koba, M. Cichocka, Cieszyn: A. Sokal, A. Kubicius, E. Pruchniewicz, Gliwice: A. Kowalik‐Sztylc, W. Czapla, Katowice: I. Mróz, M. Kozlowski, T. Pawlowski, M. Tendera, Katowice: A. Winiarska‐Filipek, A. Fidyk, A. Slowikowski, M. Haberka, M. Lachor‐Broda, M. Biedron, Z. Gasior, Kielce: M. Kołodziej, M. Janion, Kielce: I. Gorczyca‐Michta, B. Wozakowska‐Kaplon, Łódź: M. Stasiak, P. Jakubowski, T. Ciurus, J. Drozdz, Łódź: M. Simiera, P. Zajac, T. Wcislo, P. Zycinski, J. Kasprzak, Nysa: A. Olejnik, E. Harc‐Dyl, J. Miarka, M. Pasieka, M. Ziemińska‐Łuć, W. Bujak, Opoczno: A. Śliwiński, A. Grech, J. Morka, K. Petrykowska, M. Prasał, Opole: G. Hordyński, P. Feusette, P. Lipski, A. Wester, Radlin: W. Streb, Rzeszów: J. Romanek, P. Woźniak, M. Chlebuś, P. Szafarz, W. Stanik, Szczecin: M. Zakrzewski, J. Kaźmierczak, Szczecin: A. Przybylska, E. Skorek, H. Błaszczyk, M. Stępień, S. Szabowski, W. Krysiak, M. Szymańska, Tarnów: J. Karasiński, J. Blicharz, M. Skura, Warsaw: K. Hałas, L. Michalczyk, Z. Orski, K. Krzyżanowski, A. Skrobowski, Warsaw: L. Zieliński, M. Tomaszewska‐Kiecana, M. Dłużniewski, Warsaw: M. Kiliszek, M. Peller, M. Budnik, P. Balsam, G. Opolski, A. Tymińska, K. Ozierański, A. Wancerz, Warsaw: A. Borowiec, E. Majos, R. Dabrowski, H. Szwed, Zabrze: A. Musialik‐Lydka, Zabrze: A. Leopold‐Jadczyk, E. Jedrzejczyk‐Patej, M. Koziel, R. Lenarczyk, M. Mazurek, Z. Kalarus, Zabrze: K. Krzemien‐Wolska, P. Starosta, E. Nowalany‐Kozielska, Zakopane: A. Orzechowska, M. Szpot, M. Staszel, **Portugal** Almada: S. Almeida, H. Pereira, L. Brandão Alves, R. Miranda, L. Ribeiro, Carnaxide Lisboa: F. Costa, F. Morgado, P. Carmo, P. Galvao Santos, R. Bernardo, P. Adragão, Santarém: G. Ferreira da Silva, M. Peres, M. Alves, M. Leal, Vila Real: A. Cordeiro, P. Magalhães, P. Fontes, S. Leão, Viseu: A. Delgado, A. Costa, B. Marmelo, B. Rodrigues, D. Moreira, J. Santos, L. Santos, **Romania** Arad: A. Terchet, D. Darabantiu, S. Mercea, V. Turcin Halka, A. Pop Moldovan, Brasov: A. Gabor, B. Doka, G. Catanescu, H. Rus, L. Oboroceanu, E. Bobescu, Bucharest: R. Popescu, A. Dan, A. Buzea, I. Daha, G. Dan, I. Neuhoff, Bucharest: M. Baluta, R. Ploesteanu, N. Dumitrache, M. Vintila, Bucharest: A. Daraban, C. Japie, E. Badila, H. Tewelde, M. Hostiuc, S. Frunza, E. Tintea, D. Bartos, Bucharest: A. Ciobanu, I. Popescu, N. Toma, C. Gherghinescu, D. Cretu, N. Patrascu, C. Stoicescu, C. Udroiu, G. Bicescu, V. Vintila, D. Vinereanu, M. Cinteza, R. Rimbas, Iași: M. Grecu, Oradea: A. Cozma, F. Boros, M. Ille, O. Tica, R. Tor, A. Corina, A. Jeewooth, B. Maria, C. Georgiana, C. Natalia, D. Alin, D. Dinu‐Andrei, M. Livia, R. Daniela, R. Larisa, S. Umaar, T. Tamara, M. Ioachim Popescu, Târgu Mureș: D. Nistor, I. Sus, O. Coborosanu, Timișoara: N. Alina‐Ramona, R. Dan, L. Petrescu, Timișoara: G. Ionescu, I. Popescu, C. Vacarescu, E. Goanta, M. Mangea, A. Ionac, C. Mornos, D. Cozma, S. Pescariu, **RUSSIAN FEDERATION** Arkhangelsk: E. Solodovnicova, I. Soldatova, J. Shutova, L. Tjuleneva, T. Zubova, V. Uskov, Arkhangelsk: D. Obukhov, G. Rusanova, Arkhangelsk: I. Soldatova, N. Isakova, S. Odinsova, T. Arhipova, Arkhangelsk: E. Kazakevich, E. Serdechnaya, O. Zavyalova, Saint‐Petersburg: T. Novikova, Saint‐Petersburg: I. Riabaia, S. Zhigalov, Saint‐Petersburg: E. Drozdova, I. Luchkina, Y. Monogarova, Vladivostok: D. Hegya, L. Rodionova, L. Rodionova, V. Nevzorova, Vladivostok: I. Soldatova, O. Lusanova, **Serbia** Belgrade: A. Arandjelovic, D. Toncev, L. Vukmirovic, M. Radisavljevic, M. Milanov, N. Sekularac, Belgrade: M. Zdravkovic, S. Hinic, S. Dimkovic, T. Acimovic, J. Saric, S. Radovanovic, Belgrade: A. Kocijancic, B. Obrenovic‐Kircanski, D. Kalimanovska Ostric, D. Simic, I. Jovanovic, I. Petrovic, M. Polovina, M. Vukicevic, M. Tomasevic, N. Mujovic, N. Radivojevic, O. Petrovic, S. Aleksandric, V. Kovacevic, Z. Mijatovic, B. Ivanovic, M. Tesic, T. Potpara, A. Ristic, B. Vujisic‐Tesic, M. Nedeljkovic, Belgrade: A. Karadzic, A. Uscumlic, M. Prodanovic, M. Zlatar, M. Asanin, Belgrade: B. Bisenic, V. Vasic, Z. Popovic, Belgrade: D. Djikic, M. Sipic, V. Peric, B. Dejanovic, N. Milosevic, Belgrade: S. Backovic, A. Stevanovic, A. Andric, B. Pencic, M. Pavlovic‐Kleut, V. Celic, Kragujevac: M. Pavlovic, M. Petrovic, M. Vuleta, N. Petrovic, S. Simovic, Z. Savovic, S. Milanov, G. Davidovic, V. Iric‐Cupic, Niš: D. Djordjevic, M. Damjanovic, S. Zdravkovic, V. Topic, D. Stanojevic, M. Randjelovic, R. Jankovic‐Tomasevic, V. Atanaskovic, S. Antic, M. Pavlovic, Niška Banja: D. Simonovic, M. Stojanovic, S. Stojanovic, V. Mitic, V. Ilic, D. Petrovic, M. Deljanin Ilic, S. Ilic, V. Stoickov, Pirot: S. Markovic, Šabac: A. Mijatovic, D. Tanasic, D. Petrovic, G. Radakovic, J. Peranovic, M. Pavlovic, N. Panic‐Jelic, O. Vujadinovic, P. Pajic, S. Bekic, S. Kovacevic, **Spain** Alicante: A. García Fernandez, Benalmadena: A. Perez Cabeza, Córdoba: M. Anguita, Granada: L. Tercedor Sanchez, Huarte: E. Mau, J. Loayssa, M. Ayarra, M. Carpintero, Madrid: I. Roldán Rabadan, Murcia: M. Leal, Murcia: M. Gil Ortega, Murcia: A. Tello Montoliu, E. Orenes Piñero, S. Manzano Fernández, F. Marín, A. Romero Aniorte, A. Veliz Martínez, M. Quintana Giner, Pamplona: G. Ballesteros, M. Palacio, O. Alcalde, I. García‐Bolao, San Juan de Alicante: V. Bertomeu Gonzalez, Santiago de Compostela: F. Otero‐Raviña, J. García Seara, J. Gonzalez Juanatey, **Switzerland** Geneva: N. Dayal, P. Maziarski, P. Gentil‐Baron, D. Shah, **Turkey** Adana: M. Koç, Afyon: E. Onrat, I. E. Dural, Ankara: K. Yilmaz, B. Özin, Ankara: S. Tan Kurklu, Y. Atmaca, Ankara: U. Canpolat, L. Tokgozoglu, Ankara: A. K. Dolu, B. Demirtas, D. Sahin, Ankara: O. Ozcan Celebi, E. Diker, Antalya: G. Gagirci, Bayraklı/Izmir: U.O. Turk, Bursa: H. Ari, Diyarbakır: N. Polat, N. Toprak, Gaziantep: M. Sucu, Görükle‐Bursa: O. Akin Serdar, Istanbul: A. Taha Alper, Istanbul: A. Kepez, Istanbul: Y. Yuksel, Kurupelit ‐ Samsun: A. Uzunselvi, S. Yuksel, M. Sahin, Merkez/Düzce: O. Kayapinar, Mersin: T. Ozcan, Sivas: H. Kaya, M. B. Yilmaz, Trabzon: M. Kutlu, Yüreğir‐Adana: M. Demir, **United Kingdom** Barnstaple: C. Gibbs, S. Kaminskiene, M. Bryce, A. Skinner, G. Belcher, J. Hunt, L. Stancombe, B. Holbrook, C. Peters, S. Tettersell, Birmingham: A. Shantsila, D. Lane, K. Senoo, M. Proietti, K. Russell, P. Domingos, S. Hussain, J. Partridge, R. Haynes, S. Bahadur, R. Brown, S. McMahon, G. Y H Lip, Blackburn: J. McDonald, K. Balachandran, R. Singh, S. Garg, H. Desai, K. Davies, W. Goddard, Blackpool: G. Galasko, I. Rahman, Y. Chua, O. Payne, S. Preston, O. Brennan, L. Pedley, C. Whiteside, C. Dickinson, J. Brown, K. Jones, L. Benham, R. Brady, Carlisle: L. Buchanan, A. Ashton, H. Crowther, H. Fairlamb, S. Thornthwaite, C. Relph, A. McSkeane, U. Poultney, N. Kelsall, P. Rice, T. Wilson, Chertsey: M. Wrigley, R. Kaba, T. Patel, E. Young, J. Law, Cramlington: C. Runnett, H. Thomas, H. McKie, J. Fuller, S. Pick, Exeter: A. Sharp, A. Hunt, K. Thorpe, C. Hardman, E. Cusack, L. Adams, M. Hough, S. Keenan, A. Bowring, J. Watts, Great Yarmouth: J. Zaman, K. Goffin, H. Nutt, Harrogate: Y. Beerachee, J. Featherstone, C. Mills, J. Pearson, L. Stephenson, Huddersfield: S. Grant, A. Wilson, C. Hawksworth, I. Alam, M. Robinson, S. Ryan, Macclesfield: R. Egdell, E. Gibson, M. Holland, D. Leonard, Maidstone: B. Mishra, S. Ahmad, H. Randall, J. Hill, L. Reid, M. George, S. McKinley, L. Brockway, W. Milligan, Manchester: J. Sobolewska, J. Muir, L. Tuckis, L. Winstanley, P. Jacob, S. Kaye, L. Morby, Nottingham: A. Jan, T. Sewell, Poole: C. Boos, B. Wadams, C. Cope, P. Jefferey, Portsmouth: N. Andrews, A. Getty, A. Suttling, C. Turner, K. Hudson, R. Austin, S. Howe, Redhill: R. Iqbal, N. Gandhi, K. Brophy, P. Mirza, E. Willard, S. Collins, N. Ndlovu, Rhyl: E. Subkovas, V. Karthikeyan, L. Waggett, A. Wood, A. Bolger, J. Stockport, L. Evans, E. Harman, J. Starling, L. Williams, V. Saul, Salisbury: M. Sinha, L. Bell, S. Tudgay, S. Kemp, J. Brown, L. Frost, Shrewsbury: T. Ingram, A. Loughlin, C. Adams, M. Adams, F. Hurford, C. Owen, C. Miller, D. Donaldson, H. Tivenan, H. Button, South Shields: A. Nasser, O. Jhagra, B. Stidolph, C. Brown, C. Livingstone, M. Duffy, P. Madgwick, Southampton: P. Roberts, E. Greenwood, L. Fletcher, M. Beveridge, S. Earles, Taunton: D. McKenzie, D. Beacock, M. Dayer, M. Seddon, D. Greenwell, F. Luxton, F. Venn, H. Mills, J. Rewbury, K. James, K. Roberts, L. Tonks, Torquay: D. Felmeden, W. Taggu, A. Summerhayes, D. Hughes, J. Sutton, L. Felmeden, Watford: M. Khan, E. Walker, L. Norris, L. O'Donohoe, Weston‐super‐Mare: A. Mozid, H. Dymond, H. Lloyd‐Jones, G. Saunders, D. Simmons, D. Coles, D. Cotterill, S. Beech, S. Kidd, Wolverhampton: B. Wrigley, S. Petkar, A. Smallwood, R. Jones, E. Radford, S. Milgate, S. Metherell, V. Cottam, Yeovil: C. Buckley, A. Broadley, D. Wood, J. Allison, K. Rennie, L. Balian, L. Howard, L. Pippard, S. Board, T. Pitt‐Kerby.
