# Supplementary material for: Impact of renal impairment on atrial fibrillation: ESC‐EHRA EORP‐AF Long‐Term General Registry
Source: Eur J Clin Invest. 2022 Jan 17;52(6):e13745. doi: 10.1111/eci.13745 (PMC9287022; doi:10.1111/eci.13745)
Supplement: Supplementary file 1 — App S1 [file ECI-52-0-s001.docx]

Appendix S1

Impact of renal impairment on atrial fibrillation: **ESC-EHRA EORP-AF Long-Term General Registry**

Short title: Long-term AF outcomes with renal impairment

Wern Yew Ding^1^; Tatjana S. Potpara^2,3^; Carina Blomström-Lundqvist^4^; Giuseppe Boriani^5^; Francisco Marin^6^; Laurent Fauchier^7^; Gregory Y. H. Lip^1,8^; on behalf of the ESC-EHRA EORP-AF Long-Term General Registry Investigators^9^

^1^ Liverpool Centre for Cardiovascular Science, University of Liverpool and Liverpool Heart & Chest Hospital, Liverpool, United Kingdom; ^2^ School of Medicine, University of Belgrade, Belgrade, Serbia; ^3^ Intensive Arrhythmia Care, Cardiology Clinic, Clinical Center of Serbia, Belgrade, Serbia; ^4^ Department of Medical Science and Cardiology, Uppsala University, Uppsala, Sweden; ^5^ Cardiology Division, Department of Biomedical, Metabolic and Neural Sciences, University of Modena and Reggio Emilia, Policlinico di Modena, Modena, Italy; ^6^ Department of Cardiology, Hospital Universitario Virgen de la Arrixaca, IMIB-Arrixaca, University of Murcia, CIBERCV, Murcia, Spain; ^7^ Service de Cardiologie, Centre Hospitalier Universitaire Trousseau, Tours, France; ^8^ Aalborg Thrombosis Research Unit, Department of Clinical Medicine, Aalborg University, Aalborg, Denmark; ^9^ Listed in Appendix.

Corresponding author:

Prof Gregory Y H Lip [gregory.lip@liverpool.ac.uk](mailto:gregory.lip@liverpool.ac.uk)

# **Table S1**. Primary diagnosis and symptoms based on renal function at baseline admission or consultation

|  | Total  (n=9306) | | eGFR ≥90  (n=1573; 16.9%) | | eGFR 60-89  (n=4586; 49.3%) | | eGFR 30-59  (n=2790; 30.0%) | | eGFR <30  (n=357; 3.8%) | | | p value  (trend) |  |
| --- | --- | --- | --- | --- | --- | --- | --- | --- | --- | --- | --- | --- | --- |
| Primary diagnosis, n (%) |  |  |  |  |  |  |  |  |  |  | <0.001 | | |
| Acute coronary syndrome | 381 | (4.1) | 53 | (3.4) | 187 | (4.1) | 123 | (4.4) | 18 | (5.0) |  | | |
| Atrial fibrillation | 6098 | (65.5) | 1233 | (78.4) | 3162 | (68.9) | 1582 | (56.7) | 121 | (33.9) |  | | |
| Heart failure | 1086 | (11.7) | 106 | (6.7) | 426 | (9.3) | 458 | (16.4) | 96 | (26.9) |  | | |
| Hypertension | 162 | (1.7) | 18 | (1.1) | 75 | (1.6) | 61 | (2.2) | 8 | (2.2) |  | | |
| Coronary artery disease | 246 | (2.6) | 21 | (1.3) | 141 | (3.1) | 72 | (2.6) | 12 | (3.4) |  | | |
| Valvular heart disease | 277 | (3.0) | 28 | (1.8) | 138 | (3.0) | 92 | (3.3) | 19 | (5.3) |  | | |
| Other cardiovascular | 721 | (7.7) | 75 | (4.8) | 317 | (6.9) | 281 | (10.1) | 48 | (13.4) |  | | |
| Non-cardiovascular | 335 | (3.6) | 39 | (2.5) | 140 | (3.1) | 121 | (4.3) | 35 | (9.8) |  | | |
| Symptoms, n (%) |  |  |  |  |  |  |  |  |  |  |  | | |
| EHRA I | 3936 | (42.3) | 601 | (38.2) | 1953 | (42.6) | 1215 | (43.5) | 167 | (46.8) | 0.001 | | |
| EHRA II | 3391 | (36.4) | 624 | (39.7) | 1753 | (38.2) | 915 | (32.8) | 99 | (27.7) | <0.001 | | |
| EHRA III | 1755 | (18.9) | 305 | (19.4) | 801 | (17.5) | 585 | (21.0) | 64 | (17.9) | 0.002 | | |
| EHRA IV | 223 | (2.4) | 43 | (2.7) | 78 | (1.7) | 75 | (2.7) | 27 | (7.6) | <0.001 | | |

CKD, chronic kidney disease; eGFR, estimated glomerular filtration rate; EHRA, European Heart Rhythm Association.

# **Table S2**. Diagnostics and baseline interventions based on renal function

|  | Total  (n=9306) | | eGFR ≥90  (n=1573; 16.9%) | | eGFR 60-89  (n=4586; 49.3%) | | eGFR 30-59  (n=2790; 30.0%) | | eGFR <30  (n=357; 3.8%) | | p value  (trend) |
| --- | --- | --- | --- | --- | --- | --- | --- | --- | --- | --- | --- |
| Recent diagnostics, n (%) |  |  |  |  |  |  |  |  |  |  |  |
| Holter monitoring | 2064 | (22.5) | 373 | (24.1) | 1051 | (23.2) | 577 | (21.0) | 63 | (17.8) | 0.009 |
| Exercise test | 743 | (8.1) | 153 | (9.9) | 378 | (8.4) | 197 | (7.2) | 15 | (4.3) | 0.001 |
| Trans-thoracic echocardiography | 7984 | (86.4) | 1350 | (86.1) | 3914 | (85.9) | 2409 | (87.2) | 311 | (87.6) | 0.401 |
| Trans-oesophageal echocardiography | 1347 | (14.6) | 272 | (17.4) | 669 | (14.7) | 364 | (13.2) | 42 | (11.8) | 0.001 |
| Cardiac computed tomography | 415 | (4.5) | 103 | (6.6) | 204 | (4.5) | 104 | (3.8) | 4 | (1.1) | <0.001 |
| Cardiac magnetic resonance imaging | 140 | (1.5) | 32 | (2.1) | 63 | (1.4) | 41 | (1.5) | 4 | (1.1) | 0.274 |
| Electrophysiology study | 255 | (2.8) | 84 | (5.4) | 117 | (2.6) | 53 | (1.9) | 1 | (0.3) | <0.001 |
| Coronary angiography | 1401 | (15.2) | 243 | (15.6) | 694 | (15.3) | 406 | (14.8) | 58 | (16.4) | 0.815 |
| Current interventions, n (%) |  |  |  |  |  |  |  |  |  |  |  |
| Cardioversion | 2057 | (55.9) | 418 | (58.2) | 1013 | (54.4) | 574 | (58.0) | 52 | (48.6) | 0.063 |
| Electrical cardioversion | 1704 | (46.3) | 356 | (49.6) | 885 | (47.5) | 429 | (43.3) | 34 | (31.8) | 0.001 |
| Pharmacological cardioversion | 855 | (23.3) | 183 | (25.5) | 389 | (20.9) | 253 | (25.6) | 30 | (28.0) | 0.007 |
| Catheter AF ablation | 441 | (12.0) | 121 | (16.9) | 256 | (13.7) | 60 | (6.1) | 4 | (3.7) | <0.001 |

AF, atrial fibrillation; eGFR, estimated glomerular filtration rate; CKD, chronic kidney disease.

# **Table S3**. Univariate analysis on predictors of the composite outcome of thromboembolism, major bleeding, acute coronary syndrome and all-cause mortality

|  | Composite outcome of TE, major bleeding, ACS and all-cause death  Unadjusted HR (95% CI) | p value |
| --- | --- | --- |
| Age (per year) | 1.05 (1.04 - 1.05) | <0.001 |
| Female sex | 1.01 (0.87 - 1.18) | 0.880 |
| BMI (per kg/m^2^) | 0.97 (0.95 - 0.98) | <0.001 |
| eGFR (each categorical increase in CKD severity) | 1.65 (1.49 - 1.82) | <0.001 |
| eGFR (continuous, per 10 mL/min/1.73m^2^ decrease) | 1.21 (1.17 - 1.26) | <0.001 |
| LA diameter (per mm) | 1.01 (1.00 - 1.02) | 0.044 |
| LV ejection fraction (per %) | 0.98 (0.97 - 0.99) | <0.001 |
| Alcohol consumption ≥2 units/day | 0.94 (0.70 - 1.26) | 0.687 |
| Current smoker | 0.85 (0.64 - 1.12) | 0.239 |
| No regular exercise | 1.62 (1.39 - 1.90) | <0.001 |
| Any cardiomyopathy | 1.13 (0.92 - 1.39) | 0.249 |
| Congenital heart disease | 1.03 (0.52 - 2.08) | 0.925 |
| COPD | 1.70 (1.36 - 2.12) | <0.001 |
| Coronary artery disease | 2.51 (2.14 - 2.93) | <0.001 |
| Diabetes mellitus | 1.50 (1.27 - 1.77) | <0.001 |
| Heart failure | 1.97 (1.69 - 2.29) | <0.001 |
| Hypertension | 1.19 (1.01 - 1.39) | 0.036 |
| Liver disease | 1.41 (0.95 - 2.10) | 0.092 |
| Malignancy | 1.18 (1.03 - 1.36) | 0.014 |
| Peripheral vascular disease | 1.81 (1.44 - 2.27) | <0.001 |
| Previous haemorrhagic event | 1.76 (1.34 - 2.30) | <0.001 |
| Previous thromboembolic event | 1.65 (1.35 - 2.01) | <0.001 |
| Sleep apnoea | 0.95 (0.74 - 1.21) | 0.665 |
| Hyperthyroidism | 0.86 (0.46 - 1.61) | 0.647 |
| Hypothyroidism | 1.17 (0.86 - 1.59) | 0.323 |
| Valvular heart disease | 1.26 (1.08 - 1.47) | 0.003 |
| Any antiplatelet | 2.23 (1.90 - 2.62) | <0.001 |
| VKA therapy | 1.09 (0.94 - 1.25) | 0.261 |
| NOAC therapy | 0.76 (0.66 - 0.89) | <0.001 |

ACS, acute coronary syndrome; BMI, body mass index; CI, confidence interval; CKD, chronic kidney disease; COPD, chronic obstructive pulmonary disease; eGFR, estimated glomerular filtration rate; EHRA, European Heart Rhythm Association; HR, hazard ratio; LA, left atrial; LV, left ventricular; NOAC, non-vitamin K antagonist oral anticoagulant; TE, thromboembolism; VKA, vitamin K antagonist.
